# Accessory Chromosome Contributes to Virulence of Banana Infecting *Fusarium oxysporum* Tropical Race 4

**DOI:** 10.1111/mpp.70146

**Published:** 2025-09-12

**Authors:** Jelmer Dijkstra, Anouk C. van Westerhoven, Lucía Gómez‐Gil, Carolina Aguilera‐Galvez, Giuliana Nakasato‐Tagami, Sebastien D. Garnier, Masaya Yamazaki, Tsutomu Arie, Takashi Kamakura, Takayuki Arazoe, Antonio Di Pietro, Michael F. Seidl, Gert H. J. Kema

**Affiliations:** ^1^ Laboratory of Phytopathology Wageningen University and Research Wageningen the Netherlands; ^2^ Theoretical Biology & Bioinformatics Group, Department of Biology Utrecht University Utrecht the Netherlands; ^3^ Departamento de Genética Universidad de Córdoba, Campus de Excelencia Agroalimentario (ceiA3) Córdoba Spain; ^4^ Área de Sistemas Naturales y Sostenibilidad, Escuela de Ciencias Aplicadas e Ingeniería Universidad EAFIT Medellín Colombia; ^5^ Faculty of Science and Technology Tokyo University of Science Chiba Japan; ^6^ Graduate School of Agriculture Tokyo University of Agriculture and Technology (TUAT) Tokyo Japan

**Keywords:** accessory chromosome, banana, *Fusarium*, tropical race 4, virulence

## Abstract

Filamentous fungi have evolved compartmentalised genomes comprising conserved core regions and dynamic accessory regions, which are thought to drive adaptation to changing environments, including interactions with host organisms. Tropical Race 4 (TR4) is a lineage of banana‐infecting *Fusarium* spp. and causes a devastating Fusarium wilt epidemic in the industrial banana cultivar Cavendish. A recent study showed that TR4 contains a single accessory chromosome (chromosome 12), which in some strains has undergone extensive internal duplications, tripling its size compared to other closely related strains. However, the contribution of this accessory chromosome to virulence is currently unknown. Here we show that the induced loss of accessory chromosome 12 in the TR4 reference strain II5 leads to reduced virulence on banana plants. Moreover, loss of chromosome 12 co‐occurs with structural rearrangements of conserved core chromosomes. Together, our results provide new insights into the chromosome dynamics of the banana‐infecting *Fusarium* TR4 lineage and highlight the importance of its unique accessory chromosome in virulence.

## Graphical abstract

The independent accessory chromosome 12 (AC12) is dispensable for vegetative growth but contributes significantly to the virulence of banana‐infecting *Fusarium oxysporum* Tropical Race 4.

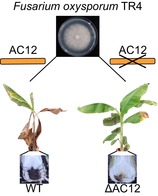

## 1 Introduction

Genome compartmentalisation with conserved core regions that are separated from dynamic accessory regions is a characteristic of numerous filamentous pathogens (Croll and McDonald 2012; Dong et al. 2015; Bertazzoni et al. 2018; Torres et al. 2020; Wacker et al. 2023). Accessory regions likely evolved to facilitate rapid adaptation to changing environments, including host interactions (Croll and McDonald 2012; Yu et al. 2023; Torres et al. 2020). These genomic regions show distinct sequence characteristics compared to their core counterparts, including higher sequence variation, abundance in transposable elements (TEs), different codon usage, and lower gene density (Coleman et al. 2009; Seidl and Thomma 2017; Habig and Stukenbrock 2020; Bertazzoni et al. 2018). Moreover, accessory regions are usually enriched for histone modifications associated with facultative heterochromatin (Connolly et al. 2013; Schotanus et al. 2015; Fokkens et al. 2018; Cook et al. 2020). Accessory regions can either be embedded in core chromosomes or comprise complete independent accessory chromosomes (Ma et al. 2010; van Westerhoven et al. 2024).

Although the possible function or impact of accessory regions is not always clear (Möller et al. 2018; Ayukawa et al. 2021), many play important roles during the interaction between fungal pathogens and plant hosts (Johnson et al. 2001; Ma et al. 2010; de Jonge et al. 2013; Plaumann et al. 2018; Ayukawa et al. 2021; Sakane et al. 2024; Wei et al. 2024), often by encoding secreted effector proteins (Bertazzoni et al. 2018; Yang et al. 2020). The presence of accessory regions or chromosomes may also facilitate horizontal transfer of beneficial traits, as natural populations from different fungal species bear signs of such horizontal transfer events (Masel 1996; Akagi et al. 2009; Habig et al. 2024; Barragan et al. 2024). Experimental horizontal chromosome transfer has been achieved in several species (Ma et al. 2010; Li, Fokkens, van Dam, and Rep 2020; van Dam et al. 2017; Habig et al. 2024; Akagi et al. 2009; He et al. 1998). However, despite these advances, many questions on the origin and evolution of accessory regions remain unanswered (Habig and Stukenbrock 2020).

Members of the *Fusarium oxysporum* species complex (FOSC) contain lineage‐specific accessory regions, which display characteristics common to analogous genomic regions in other fungi (Yang et al. 2020; van Westerhoven et al. 2024), including the presence of effector genes that determine host‐specific virulence (Ma et al. 2010; Li, Fokkens, Conneely, and Rep 2020; Li, Fokkens, van Dam, and Rep 2020; Ayukawa et al. 2021; Sakane et al. 2024). Furthermore, horizontal acquisition of an accessory chromosome by a nonpathogenic strain can turn it into a plant pathogen (Ma et al. 2010). Importantly, accessory effector profiles are often shared between lineages infecting the same or a similar host (van Dam et al. 2016; Brenes Guallar et al. 2022). Loss of accessory chromosomes has been experimentally induced in several *F. oxysporum* strains (Ayukawa et al. 2021; Sakane et al. 2024; van Dam et al. 2017). For example, induced loss of a small accessory chromosome in *F. oxysporum* f. sp. *radicis‐cucuminerum* resulted in complete loss of virulence on cucurbit hosts (van Dam et al. 2017).

The causal agents of Fusarium wilt of banana (FWB) are separated into three different races based on their pathogenicity to different banana cultivars (Ploetz 2015) and contain a diverse set of accessory regions (van Westerhoven et al. 2024). The current devastating FWB epidemic is caused by the tropical race 4 (TR4) that can infect several banana varieties, including Cavendish, which is widely used by the banana industry (Ordóñez et al. 2015). TR4 isolates are highly similar to each other and have recently been reclassified into a new species, *F. odoratissimum* (Maryani et al. 2019). The TR4 reference strain II5 contains two large accessory regions; one is part of core chromosome 1 and the other is an independent chromosome named AC12 (van Westerhoven et al. 2024; Dijkstra et al. 2024). Previous work provided evidence for large‐scale duplications in the AC12 sequence of a subset of sequenced TR4 strains, including strain II5 (van Westerhoven et al. 2024, Dijkstra et al. 2024). Comparison between the TR4 strains II5 and M1 (van Westerhoven et al. 2023), the latter lacking the large‐scale duplications in the AC12 sequence (Dijkstra et al. 2024), revealed their overall similarity both in the accessory and core regions (Dijkstra et al. 2024; van Westerhoven et al. 2024).

Given the importance of TR4 for global banana production, these findings raise important questions about the evolution of AC12 and its potential contribution to adaptation and virulence. To analyse the role of AC12 and the phenotypic effect of chromosomal changes in *F. oxysporum* strain II5, we generated AC12 loss mutants through benomyl treatment and tested the newly generated mutants for different growth and virulence‐related traits. We show that AC12 is dispensable for vegetative growth but contributes to virulence on banana plants and tolerance to abiotic stresses. Together, these results highlight the relevance of AC12 in niche adaptation of the TR4 lineage.

## 2 Results

### 2.1 Accessory Chromosome 12 Is Dispensable for Vegetative Growth

**FIGURE 1:**
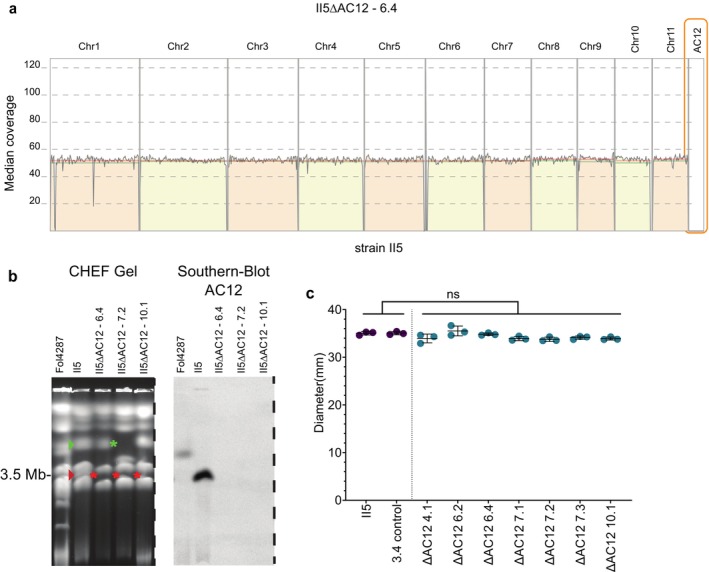
Accessory chromosome 12 (AC12) is dispensable for vegetative growth of II5. (a) Mapping of Illumina short reads from the II5ΔAC12 mutant 6.4 to the reference genome assembly of strain II5 (van Westerhoven et al. 2024). Note the specific loss of sequences mapping to AC12. (b) CHEF gel showing separation of chromosomes from *Fusarium oxysporum* strains *Fol*4287 and II5, and from three independent chromosome loss mutants II5ΔAC12 6.4, II5ΔAC12 7.2, and II5ΔAC12 10.1. Note the absence of the AC12 chromosome band (left panel, red arrow and asterisks) and of corresponding hybridisation signal in the Southern blot (right panel) in the three mutants. Green arrow and asterisk indicate the position of a core chromosome that has undergone a rearrangement in mutant 7.2. The complete uncropped gel is shown in Figure S7. (c) Colony diameter (mm) of TR4 strain II5, its benomyl‐treated control 3.4, and seven independent AC12 mutants grown on potato dextrose agar plates for 6 days. No significant differences were detected between strains (Tukey–Kramer test; *p* < 0.05). Data are shown as mean ± SD (*n* = 3).

To investigate the function of AC12 in the TR4 reference strain II5, we generated chromosome loss mutants by subjecting a newly generated transformant carrying a hygromycin resistance cassette inserted in the *SIX13* locus located on AC12 (II5:Δ*SIX13*) to treatment with benomyl. Seven independent colonies that had lost hygromycin resistance were obtained, and loss of AC12 was initially confirmed by PCR with AC12‐specific primers (Figure S1). Genome sequencing and mapping of the short reads to the II5 reference genome assembly (van Westerhoven et al. 2024) confirmed the complete loss of AC12 in all seven mutants. Importantly, no other large‐scale deletions were detected after benomyl treatment (Figure 1a), resulting in similar read mapping as the wild‐type strain except for complete loss of AC12 (Figure S2). Finally, contour‐clamped homogeneous electric field (CHEF) electrophoresis followed by Southern blot analysis with an AC12‐specific probe confirmed the absence of a chromosome band of approximately 3.5 Mb (Figure 1b), the expected size of AC12 in the TR4 reference strain II5 (Dijkstra et al. 2024), in the three tested mutant strains.

Growth of the AC12 loss mutants on potato dextrose agar (PDA) plates was similar to the parental strain (Figure 1c), indicating that AC12 is dispensable for vegetative growth under these conditions. Furthermore, loss of AC12 did not impair vegetative compatibility with II5 as shown by the ability for the formation of stable heterokaryons between *nit* mutants of strain II5 and AC12 loss mutant 7.2 (Figure S3).

### 2.2 Interchromosomal Rearrangements of Core Chromosomes Occur Upon Benomyl Treatment

**FIGURE 2:**
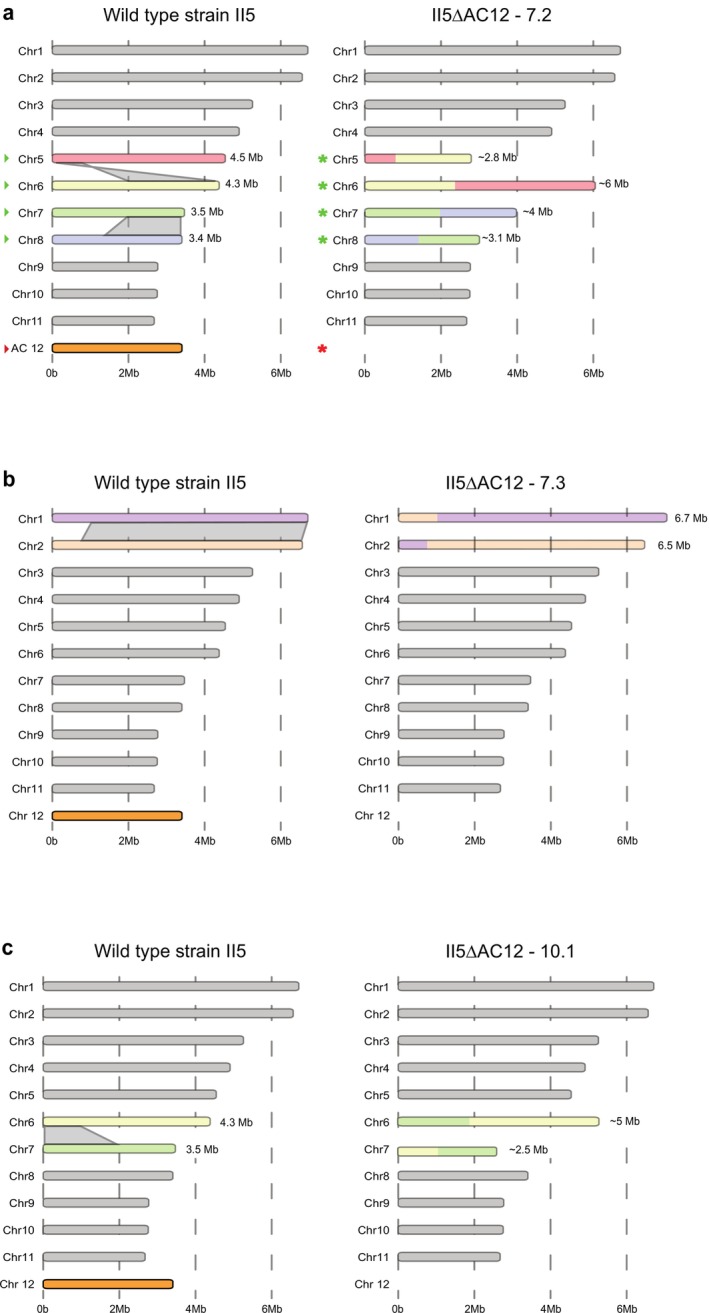
Interchromosomal rearrangements of core chromosomes following benomyl treatment and loss of accessory chromosome 12 (AC12). Schematic representation of the interchromosomal rearrangements that occurred in (a) AC12 loss mutants II5ΔAC12—7.2, (b) II5ΔAC12—7.3, and (c) II5ΔAC12—10.1. Asterisks and red arrow refer to the CHEF gel in Figure 1b. Translocations between chromosomes are indicated by grey connections and the coloured chromosome sections.

Besides the loss of AC12, CHEF gel analysis revealed additional karyotypic differences, most notably in mutant 7.2, which showed clear changes in the core chromosome electrophoretic karyotype (Figure 1b). Because sequencing did not detect any larger loss of genetic material other than AC12, we hypothesised that the difference in chromosome size observed in this mutant could be the result of interchromosomal translocations (Figure 1a). Further analysis of genome assemblies based on the short‐read sequencing data uncovered several structural changes, including small deletions (6–12 deletions per mutant) and translocations (4–10 rearrangements per mutant) (Figure S4). Most notably, five of the seven mutants showed interchromosomal rearrangements between chromosomes 7 and 8. Moreover, we detected rearrangements between chromosomes 1 and 2 in mutant 7.3 (Figure 2b); between chromosomes 5 and 6 in mutant 7.2 (Figure 2a); and between chromosomes 6 and 7 in mutant 10.1 (Figure 2c). The rearrangement between chromosomes 5 and 6 in mutant 7.2 was independently confirmed by PCR with chromosome‐specific primers (Figure S5). None of these interchromosomal rearrangements between core chromosomes caused detectable defects in vegetative growth on PDA plates (Figure 1c). In summary, these results suggest that benomyl treatment triggers interchromosomal rearrangements between core chromosomes in addition to loss of the AC12 in TR4 strain II5 and emphasise that none of these structural changes affects vegetative growth of the fungus.

Some accessory chromosomes can be horizontally transferred between different *F. oxysporum* strains through vegetative hyphal fusion (Ma et al. 2010; van Dam et al. 2017; Ayukawa et al. 2021). Attempts to transfer AC12 carrying the hygromycin resistance cassette from strain II5 to either the race 1 reference strain CR1.1 or the nonpathogenic *F. oxysporum* strain *Fo*47 (both carrying nourseothricin resistance) during co‐cultivation (30 plates tested) failed to produce double‐resistant progeny, suggesting the absence of horizontal transfer of this accessory chromosome.

### 2.3 Accessory Chromosome 12 Contributes to Hyperosmotic and Cell Wall Stress Tolerance

**FIGURE 3:**
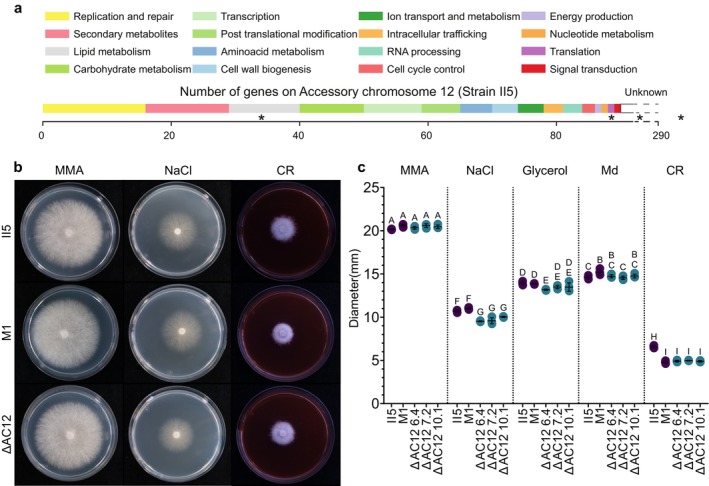
Accessory chromosome 12 (AC12) contributes to tolerance against different stresses. (a) Predicted functional categories of the genes located on AC12. Most of the genes (200) are of unknown function. Asterisks indicate gene categories significantly enriched on AC12 compared to the rest of the genome (Fisher exact test, *p* < 0.05). (b) MMA plates alone or supplemented with 0.8 M NaCl or 40 μg/mL Congo Red (CR) were inoculated with TR4 strains (multiple AC12 copies), M1 (single copy), or II5ΔAC12 6.4 (no copy) and photographed at 5 days post‐inoculation (dpi). (c) Colony diameter (mm) of TR4 strains II5, M1 or three independent AC12 loss mutants grown of MMA plates alone or supplemented with 0.8 M NaCl, 1.2 M glycerol, 10 μg/mL menadione (Md) or 40 μg/mL Congo Red (CR). Colony growth was quantified at 3 dpi. Different letters indicate significant differences between treatments (Tukey–Kramer test; *p* < 0.05). Data are shown as mean ± SD (*n* = 4).

Several of the genes located on AC12 have predicted functions in tolerance to different environmental stresses such as high osmolarity or cell wall stress (Figure 3a). For example, gene *12g01480* is related to MSN1 (Rep et al. 1999), alternatively annotated as high‐osmolarity‐induced transcription protein (Genbank: EMT69250.1). Similarly, gene *12g01620* (NCBI: XP_031052124) is related to MTL1 (Vilella et al. 2005). To test whether loss or intrachromosomal duplications of AC12 affect stress response, strains II5 (intrachromosomal duplications), M1 (no duplications), or three AC12 loss mutants (no AC12) were grown under different stress conditions. Loss of AC12 did not affect growth in minimal medium alone or supplemented with either glycerol or menadione (Figure 3b,c). However, growth at high salt concentrations (0.8 M NaCl) or in the presence of the cell wall stressor Congo Red was slightly reduced in the AC12 mutants. Similarly, strain M1 showed slightly reduced growth in the presence of Congo Red compared to II5, which could be the result of a lower AC12 gene dosage or other presence‐absence variations (van Westerhoven et al. 2024; Dijkstra et al. 2024). Both M1 and AC12 mutants showed a smoother colony edge in media containing Congo Red than II5 (Figure 3b). Overall, these results suggest that AC12 plays a role, albeit minor, in tolerance to different environmental stress factors.

### 2.4 Accessory Chromosome 12 Contributes to Virulence of TR4 on Banana Plants

**FIGURE 4:**
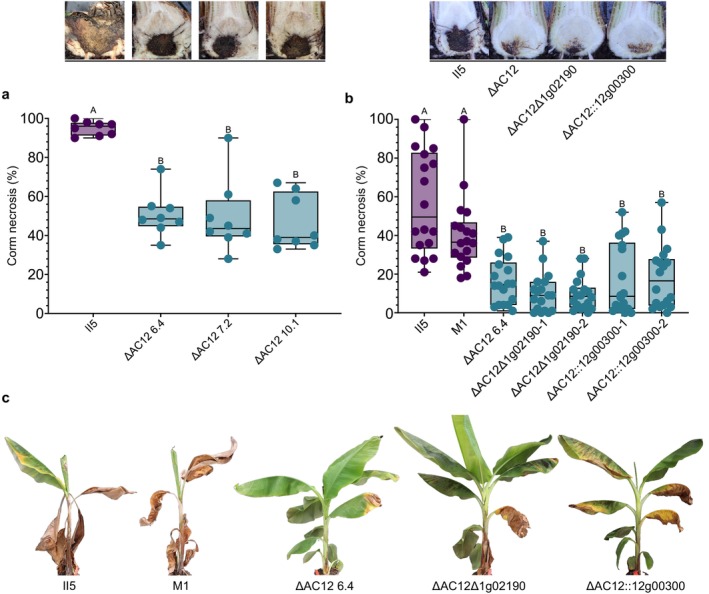
Accessory chromosome 12 (AC12) is important for virulence of II5 on Cavendish plants. (a) Percentage of corm necrosis of Cavendish ‘Grand Naine’ plants at 10 weeks post‐inoculation with reference strain II5 or with three independent AC12 loss mutants. Corm necrosis was quantified using ImageJ (*n* = 8). Representative pictures of infected corms are shown above. (b) Percentage of corm necrosis of Cavendish ‘Grand Naine’ plants inoculated with TR4 strains II5 or M1; with two independent knockout mutants in the effector gene *1g02190* obtained in the II5 ΔAC12 background (ΔAC12Δ1g02190‐1 and ΔAC12Δ1g02190‐2); or with two independent strains where the *12g00300* gene encoding an AC12‐specific effector candidate was re‐introduced into an ΔAC12 background (ΔAC12::12g0300‐1 and ΔAC12::12g0300‐2) (*n* = 18). Different letters indicate significant differences between treatments (Tukey–Kramer test; *p* < 0.05). Representative pictures of infected corms are shown above. (c) Photos showing external symptoms of representative plants analysed in panel b.

To determine the role of AC12 in virulence of TR4 on banana, plants of cultivar Cavendish were inoculated with the parental strain II5 or with three AC12 loss mutants. Ten weeks after inoculation, the extent of corm necrosis, a well‐established indicator for virulence (García‐Bastidas et al. 2019) was significantly lower (*p* < 0.05; Tukey–Kramer test) in the plants infected with the AC12 loss mutants compared to those infected with the parental strain (Figure 4a). Although the mutants were still able to infect Cavendish, the average severity of corm necrosis was 48.9% compared to 95% in the parental. Importantly, a strain treated with benomyl that had not lost AC12 caused similar corm necrosis levels as the parental strain (Figure S6). Furthermore, wilt symptoms were more severe and developed faster in the plants inoculated with II5 or M1 compared to those inoculated with the AC12 loss mutant (Figure 4c).

Secreted effectors encoded on accessory regions can act as virulence factors of TR4 against banana (Widinugraheni et al. 2018; An et al. 2019). However, the study of certain effector genes has been hampered by the fact that they are present in multiple copies in the accessory region attached to chromosome 1 and in the segmental duplications present on AC12 (van Westerhoven et al. 2024, Dijkstra et al. 2024). Hence, the availability of mutants lacking AC12 facilitates the genetic analysis of these effectors by knockout generation of the single remaining gene copy on the accessory region of chromosome 1. We obtained two independent knockout mutants of the gene *1g02190*, encoding an *in planta* upregulated (van Westerhoven et al. 2024) effector candidate located in the accessory region of chromosome 1. These mutants were generated in an ΔAC12 background, which already lacks the *1g02190* copy located on AC12 (ΔAC12Δ1g02190‐1 and ΔAC12Δ1g02190‐2). These knockout mutants did not show any additional reduction in virulence compared to the ΔAC12 mutant, suggesting that effector *1g02190* is not essential for virulence (Figure 4b,c). On the other hand, ectopic re‐introduction of *12g00300*, the only in planta upregulated effector gene uniquely encoded on AC12 (van Westerhoven et al. 2024), into an ΔAC12 loss mutant did not significantly increase levels of corm necrosis and wilt symptoms compared to the ΔAC12 mutant (Figure 4b,c). Finally, strains M1 (no AC12 intrachromosomal duplications) and II5 (AC12 intrachromosomal duplications) were equally virulent, indicating that intrachromosomal duplications of AC12 do not impact virulence under greenhouse conditions.

## 3 Discussion

Genome compartmentalisation into core and accessory regions occurs in many filamentous fungal pathogens (Dong et al. 2015; Torres et al. 2020; Wacker et al. 2023; Sánchez‐Vallet et al. 2018). Accessory genomic regions show higher genetic variation compared to the core genome, which is thought to contribute to fast adaptation (Dong et al. 2015; Torres et al. 2020), and often contain effector genes important for infection of the host (Sánchez‐Vallet et al. 2018). In different *F. oxysporum* pathosystems, accessory regions confer host‐specific virulence (Ma et al. 2010; Ayukawa et al. 2021; van Dam et al. 2017), exemplified by their ability to horizontally transfer pathogenicity to nonpathogenic isolates (Ma et al. 2010; van Dam et al. 2017; Li, Fokkens, van Dam, and Rep 2020; Ayukawa et al. 2021).

Here we investigated the role of accessory chromosome AC12 in the banana‐infecting TR4 strain II5. The viability of mutants lacking the complete AC12 chromosome demonstrates that it is dispensable for vegetative growth, as previously shown for accessory chromosomes in *F. oxysporum* strains infecting different host plants (Ma et al. 2010, Ayukawa et al. 2021, van Dam et al. 2017). Importantly, we observed a significant reduction in disease severity on banana plants inoculated with the AC12 loss mutants, suggesting an important role of this accessory chromosome in plant infection, similar to analogous accessory chromosomes in other *F. oxysporum* strains infecting different plant hosts (Li, Fokkens, Conneely, and Rep 2020; Li, Fokkens, van Dam, and Rep 2020; Ayukawa et al. 2021; Sakane et al. 2024). Furthermore, AC12 may have a broader role beyond virulence, as indicated by the finding that growth of the AC12 loss mutants was slightly impaired under high salt and cell wall stresses, and considering the predicted functions of several AC12‐encoded genes other than effectors.

In contrast to previous reports with accessory chromosome loss mutants in other *F. oxysporum formae speciales* (Ma et al. 2010; van Dam et al. 2017), the AC12 loss mutants of TR4 were still able to infect banana plants and cause characteristic FWB symptoms, albeit with reduced severity. This could be explained by the presence of an additional accessory region in isolate II5, which is attached to core chromosome 1 (van Westerhoven et al. 2024) and encodes seven predicted homologues of *SIX* effectors as well as 33 additional candidate effectors (Widinugraheni et al. 2018; An et al. 2019). Moreover, the accessory region on chromosome 1 and AC12 share many duplications, including several putative effector genes (van Westerhoven et al. 2024). Because these duplications hamper the knockout analysis of effector candidates, the newly generated AC12 loss mutants represent a useful tool to determine in the future the minimal set of effectors required to infect banana and facilitate the search for potential avirulence genes using resistant banana genotypes. To quantify the virulence contribution of AC12 and the accessory region of chromosome 1, we functionally analysed two different effector genes in the AC12 loss background. However, targeted knockout of an additional effector gene on chromosome 1 did not further reduce the virulence of the AC12 loss mutant. Similarly, re‐introduction of a single AC12‐specific effector gene into the loss mutant had no significant effects on virulence. Because the specific contribution of AC12 to the virulence of TR4 is remarkable considering the extent of regions shared with the accessory region of chromosome 1 (van Westerhoven et al. 2024), we speculate that additional AC12‐encoded genes, other than effectors, might play a more prominent role in the virulence phenotype of the chromosome loss mutants.

Besides loss of the accessory chromosome AC12, several mutant lines showed interchromosomal rearrangements of core chromosomes. These rearrangements are of interest considering the high level of collinearity commonly observed between core chromosomes of *F. oxysporum* strains (Armitage et al. 2018; Zhang et al. 2020; Kanapin et al. 2020; van Westerhoven et al. 2024). Although chromosomal rearrangements can have a strong impact both in vitro and in planta (Forche et al. 2009; Faino et al. 2016; Mehrabi et al. 2017; Seidl and Thomma 2014; Olarte et al. 2019), we observed no phenotypic differences in the mutants that could be associated with these core chromosome rearrangements. Additionally, AC12 in strain II5 contains a high number of duplications, including a near triplication of the chromosome size likely due to intrachromosomal duplications (van Westerhoven et al. 2024; Dijkstra et al. 2024). We failed to detect a significant difference in growth rate or virulence between TR4 strains II5 and M1, which do or do not carry the intrachromosomal duplications of AC12, respectively. It has been suggested that duplications of accessory genetic material might be a prelude to rapid sequence divergence and effector diversification (Seong and Krasileva 2023). To date, however, a clear fitness effect linked to the duplications of AC12 remains to be discovered, because TR4 strains with or without the duplications show similar virulence levels.

In summary, our results confirm the presence of intrachromosomal duplications of the independent accessory chromosome AC12 in the TR4 reference strain II5 and demonstrate its contribution to different biological processes, particularly plant infection. The observed intrachromosomal duplications and chromosomal rearrangements indicate a high degree of genome plasticity, highlighting an important mechanism of accessory chromosome diversification in fungal plant pathogens. The implications of these adaptations on the virulence and/or biology of *F. oxysporum* remain to be determined. Further knowledge on the function and dynamics of accessory regions in the TR4 lineage will be crucial for combating this devastating banana pathogen.

## 4 Experimental Procedures

### 4.1 Fungal Growth Conditions and Chromosome Loss Induction

*Fusarium oxysporum* tropical race 4 (TR4) strains II5 and M1 (van Westerhoven et al. 2023, 2024) and newly generated II5 mutant strains were routinely cultured on PDA plates at 25°C. Spore suspensions of all strains were stored as glycerol stocks at −80°C.

Chromosome loss was induced as described previously with slight modifications (Ayukawa et al. 2021). Strain II5:Δ*SIX13* was cultured in M100 medium supplemented with 25 μg/mL benomyl at 150 rpm and 25°C for 4 days. Conidia were obtained by filtering the culture through two layers of sterile cheesecloth. Conidia were plated on M100 plates supplemented with 0.04% Triton X‐100. Plates were overlaid with a piece of sterile filter paper and incubated at 25°C for 2 to 3 days. Subsequently, the filter paper was transferred to a PDA plate with 100 μg/mL hygromycin that was incubated at 25°C for 1 day. Afterwards, the filter paper was removed, and colonies were selected for loss of hygromycin resistance by comparison to the original M100 plate. Putative chromosome loss mutants were transferred to separate PDA plates for further validation. PCR genotyping for the presence of AC12 was performed using the Phire Plant Direct PCR Master Mix (Thermo Scientific) using mycelium of putative mutants. Presence or absence of AC12 in benomyl‐treated putative mutants was tested using five different primer pairs with annealing sites spread over the length of the chromosome and compared to strain II5 used as a positive control. The chromosomal rearrangement between chromosomes 5 and 6 in strain ∆AC12 7.2 was also validated by PCR. The primers used are listed in Table S1.

### 4.2 Horizontal Chromosome Transfer

Horizontal transfer of AC12 by cocultivation of hygromycin‐resistant II5:Δ*SIX13* and nourseothricin‐resistant race 1 strain CR1.1 or nonpathogenic *F. oxysporum* strain *Fo*47 was attempted as described previously (Ayukawa et al. 2021). In brief, hygromycin‐resistant II5:Δ*SIX13* spores were spread on PDA plates in a 1:1 ratio with spores (1 × 10^5^) of either race 1 strain CR1.1 or *Fo*47. After 7 days of cocultivation at 25°C, spores were harvested and used to inoculate PDA plates containing both hygromycin (100 μg/mL) and nourseothricin (50 μg/mL). Plates were observed for the formation of double resistant colonies for 10 days.

### 4.3 Sequencing and Sequence Analysis

To validate that the mutants generated by benomyl treatment lost AC12 and analyse the effect of benomyl treatment on the presence of other genomic regions, seven AC12 mutants were sequenced. The mutants were grown in potato dextrose broth for 4 days at 150 rpm and 25°C to obtain mycelium. Cultures were centrifuged, and mycelium was freeze‐dried. DNA was extracted from freeze‐dried mycelium using the Masterpure Yeast DNA Purification Kit (LGC Biosearch Technologies). Extracted DNA was sent for Illumina whole‐genome resequencing by Beijing Genomics Institute. The reads were mapped against the II5 reference genome assembly (van Westerhoven et al. 2024) using BWA mem v. 0.7.17 (Li and Durbin 2009). The mapping was visualised using WGS coverage plotter from jvarkit v. 589510ae3 (Lindenbaum 2015).

Additionally, the short reads were assembled using SPAdes v. 3.13.0 (Bankevich et al. 2012). These assemblies were used to analyse structural variants, including chromosomal rearrangements, using mummer v. 4 (Marçais et al. 2018) followed by MUMandCO v. 3.8 (O'Donnell and Fischer 2020). Assembly statistics of the AC12 loss mutants are noted in Table S2.

To determine the putative function of the genes located on AC12, we used eggnog mapper v. 2.1.12 (Cantalapiedra et al. 2021). Putative effector genes were identified using effectorP v. 3 (Sperschneider and Dodds 2022) on the secretome determined by signalP v. 5 (Almagro Armenteros et al. 2019). Secreted in Xylem (*SIX*) genes were identified based on homology searches of *SIX* genes from *Fol*4287 using blastP v. 2.14.0 (Altschul et al. 1990).

### 4.4 CHEF Electrophoresis and Southern Blotting

Chromosomes of all strains were separated by CHEF electrophoresis and subsequent Southern blotting as described previously (Dijkstra et al. 2024).

### 4.5 Stress Tolerance Tests

Based on the functional gene annotation, different environmental stress factors were selected, and the response of AC12 mutants to these different environmental stress factors was determined. To test for osmotic stress response, 0.8 M NaCl or 1.2 M glycerol was added, whereas for the response to reactive oxygen species or cell wall stress, we added 10 μg/mL menadione or 40 μg/mL Congo Red, respectively. Strains II5, M1, and selected mutants were inoculated on PDA plates from glycerol stock. Afterwards, agar plugs from the edge of the colony were placed on regular PDA plates, MMA plates (Puhalla 1985) or MMA supplemented with either 0.8 M NaCl, 1.2 M glycerol, 10 μg/mL menadione, or 40 μg/mL Congo Red. Colony diameter was measured at 3 days post‐inoculation (dpi). Colonies were photographed at 5 dpi.

### 4.6 Vegetative Compatibility Testing

Vegetative compatibility testing was performed as described previously using potassium chlorate generated *nit* mutants of strain II5 and II5ΔAC12 7.2 (Puhalla 1985). The generated *nit* mutants were tested for compatibility on MMA plates and observed for the formation of dense hyphal growth at colony contact points, indicative of heterokaryon formation.

### 4.7 *Fusarium* Transformation

Modified CRISPR‐Cas9 vectors were used for transformation based on the plasmid previously described (Shinkado et al. 2022). This plasmid (CRISPR/Cas9‐FoU6‐FoNLSx2) contains an additional H2B nuclear localisation signal (NLS) and an optimised gRNA scaffold (Chen et al. 2013). Cloning of the target sequence into the vector and generation of the donor template were performed as previously described (Shinkado et al. 2022). TR4 reference strain II5 and II5 ΔAC12 6.4 were transformed as described (Shinkado et al. 2022) with modifications. Spores of II5 and one AC12 loss mutant (II5 ΔAC12 6.4) were produced as described for the infection assays. Subsequently, 500 μL of spore suspension was used to inoculate 50 mL of Fries medium and left to grow in the dark at 135 rpm and 20°C for 1 day. Newly formed mycelium was blended using an IKA Ultra Turrax Tube Drive P control (IKA) for 10 s at 6000 rpm. All the blended mycelium was used to inoculate 200 mL of fresh Fries medium and incubated overnight in the dark at 135 rpm and 20°C. Overnight formed mycelium was filtered through a layer of nylon mesh (20 μm) and washed three times with 50 mL of KC solution (0.6 M KCl, 65 mM CaCl_2_). Washed mycelium was collected and treated with 10 mL of the filter‐sterilised protoplasting enzyme mix consisting of 25 mg/mL driselase from *Basidiomycetes* sp., 5 mg/mL lysing enzyme from *Trichoderma harzianum*, and 100 μg/mL chitinase from *Trichoderma viride* (all obtained from Sigma‐Aldrich) in KC. The enzyme mix was incubated at 28°C with slow rocking movement for 2 h. Protoplasts were separated from the mycelium by filtering through a layer of nylon mesh. Protoplasts were centrifuged at 1800 *g* for 10 min at 4°C and washed twice using ice cold STC (1.2 M sorbitol, 10 mM Tris–HCl pH 8, 20 mM CaCl_2_). After washing, protoplasts were resuspended in 100 μL of STC per reaction. Protoplasts were then mixed with approximately 15 μg of Cas9 plasmid and 15 μg of donor template, and 100 μL of 60% PEG solution (polyethylene glycol 4000, dissolved in STC). Two different gRNA plasmids were combined for the knockout of *1g02190*. The mixture was left at room temperature for 20 min. Protoplasts were washed one last time with 1 mL of STC. Protoplasts were plated by mixing with cooled RM (34.2% sucrose, 0.1% yeast extract, 0.8% agar). After solidifying, a second layer of YG medium (2% glucose, 0.5% yeast extract, 1.5% agar) supplemented with 50 μg/mL nourseothricin was poured over the first agar layer. Resistant colonies were put on separate plates between 3 and 5 days and used for further genotyping by PCR and amplicon sequencing. Complementation of *12g00300* was performed with the same procedure without Cas9 for ectopic integration.

A II5:Δ*SIX13* mutant containing a hygromycin resistance cassette was generated by a protocol using Cas9 ribonucleoprotein gene editing as described (Wang et al. 2018) with modifications. Specifically, the NLS of H2B was cloned from TR4 strain II5 and used to replace the SV40 NLS in the Cas9 plasmid pET‐28b‐Cas9‐His (Gagnon et al. 2014). A hygromycin cassette donor template with a short 60 bp homologous overlap was used instead of larger homology arms. Protoplast plating was performed as described above. Used primers are listed in Table S1. Coding sequences and amino acid sequences of the effectors targeted for deletion or complementation are listed in Table S3.

### 4.8 Plant Growth Conditions and Infection Assays

Cavendish ‘Grand Naine’ plants were grown in the greenhouse at a day/night temperature of 25°C/23°C and a day length of 16 h with 80% relative humidity.

Infection assays of Cavendish ‘Grand Naine’ banana plants were performed as described previously (García‐Bastidas et al. 2019). In brief, strains II5, M1, and selected mutants were inoculated on PDA plates. Agar plugs from PDA plates were used to inoculate Erlenmeyer flasks containing mung bean broth. Flasks were cultivated in a rotary shaker at 150 rpm, 25°C for 5 days. Spores were collected by filtering the culture through two layers of sterile cheesecloth. Spore suspensions were subsequently diluted to 1 × 10^6^ spores per mL. Roots of the plants were injured prior to inoculation. Lastly, 200 mL of spore suspension was poured directly on the soil for each plant. Corm necrosis was quantified at 8 to 10 weeks after inoculation using ImageJ.

## Author Contributions

J.D. and A.C.W. collected data and performed the analyses. L.G.‐G., C.A.‐G., G.N.‐T., S.D.G., M.Y., T.A., T.K., and T.A. contributed to data collection and experimental methods. J.D. and A.C.W. wrote the manuscript with input from A.D.P., G.H.J.K. and M.F.S. All authors contributed to writing and editing the manuscript. G.H.J.K. and M.F.S. conceived and supervised the project. J.D. and A.C.W., as well as G.H.J.K. and M.F.S. contributed equally to this work.

## Conflicts of Interest

The authors declare no conflicts of interest.

## Supporting information


**Figure S1:** PCR analysis indicates loss of accessory chromosome 12 (AC12) after benomyl treatment. (a) Schematic representation of relative primer locations for five different AC12 PCR markers (black bands). Note that marker 1 also binds to the accessory region attached to chromosome 1. (b) Gel electrophoresis of PCR products from the parental strain II5 and putative AC12 loss mutants. Seven hygromycin‐sensitive colonies lack the bands for AC12‐specific PCR markers 2–5, but still show the band for the nonspecific marker 1.

**Figure S2:** Mapping of short reads to the II5 reference genome assembly. Mapping of Illumina reads from the parental strain II5 to the II5 reference genome assembly. Note the increased coverage at accessory chromosome 12 (AC12), indicative of intrachromosomal duplications.

**Figure S3:** AC12 mutant is vegetatively compatible with the II5 wild‐type (WT) strain. MMA plate inoculated with a *nit* mutant of the parental strain II5 and with a *nit* mutant of II5ΔAC12 7.2. Exact nitrate utilisation mutations undetermined. Annotations A and B were arbitrarily assigned to *nit* mutants with compatible mutations. Dense hyphal growth at the colony contact points indicates the formation of a nitrate‐utilising heterokaryon through vegetative compatibility.

**Figure S4:** Structural variants detected in the accessory chromosome 12 (AC12) deletion mutants. (a) The number of structural variants per variant type is shown for the seven independent AC12 deletion mutants. (b) Sizes (in bp) of the identified structural variants for the seven independent AC12 deletion mutants. Translocation sizes could not be calculated accurately due to the fragmented short read assemblies wherein translocations can be located at contig breakpoints.

**Figure S5:** Confirmation of core chromosome rearrangements in accessory chromosome 12 (AC12) loss mutant II5ΔAC12 by PCR. (a) Schematic representation of the primer locations on core chromosomes 5 and 6 in the II5 ancestor strain and the II5ΔAC12–7.2 mutant. (b) Gel electrophoresis shows PCR products from II5 and from three independent ΔAC12 mutants using different combinations of primers specific for chromosome 5 (F/R) and 6 (F/R) (see a).

**Figure S6:** Benomyl treatment does not affect virulence of TR4 strain II5. Percentage of corm necrosis of Cavendish ‘Grand Naine’ plants inoculated with the parental strain II5, benomyl treated control strain 3.4 and the chromosome loss strain II5ΔAC12 6.4. Corm necrosis was quantified using ImageJ (*n* = 10). Letters indicate significant differences between treatments (Tukey–Kramer test; *p* < 0.05).

**Figure S7:** Full uncropped CHEF gel and Southern blot used in Figure 1.

**Table S1:** Primers used in this study.

**Table S2:** Assembly statistics of the generated accessory chromosome 12 (AC12) loss mutants.

**Table S3:** Sequence information of the effectors targeted for deletion or complementation.

## Data Availability

The data that support the findings of this study are openly available in the Sequence Read Archive at https://www.ncbi.nlm.nih.gov/sra. The data sequenced in this study are available under accession no.: PRJNA1190342.
