# Supplementary material for: Accessory Chromosome Contributes to Virulence of Banana Infecting Fusarium oxysporum Tropical Race 4
Source: Mol Plant Pathol. 2025 Sep 12;26(9):e70146. doi: 10.1111/mpp.70146 (PMC12430104; doi:10.1111/mpp.70146)
Supplement: Supplementary file 1 — Figure S1: PCR analysis indicates loss of accessory chromosome 12 (AC12) after benomyl treatment. (a) Schematic representation of relative primer locations for five different AC12 PCR markers (black bands). Note that marker 1 also binds to the accessory region attached to chromosome 1. (b) Gel electrophoresis of PCR products from the parental strain II5 and putative AC12 loss mutants. Seven hygromycin‐sensitive colonies lack the bands for AC12‐specific PCR markers 2–5, but still show the band for the nonspecific marker 1. [file MPP-26-e70146-s009.docx]

**Supplementary Figures: S1**


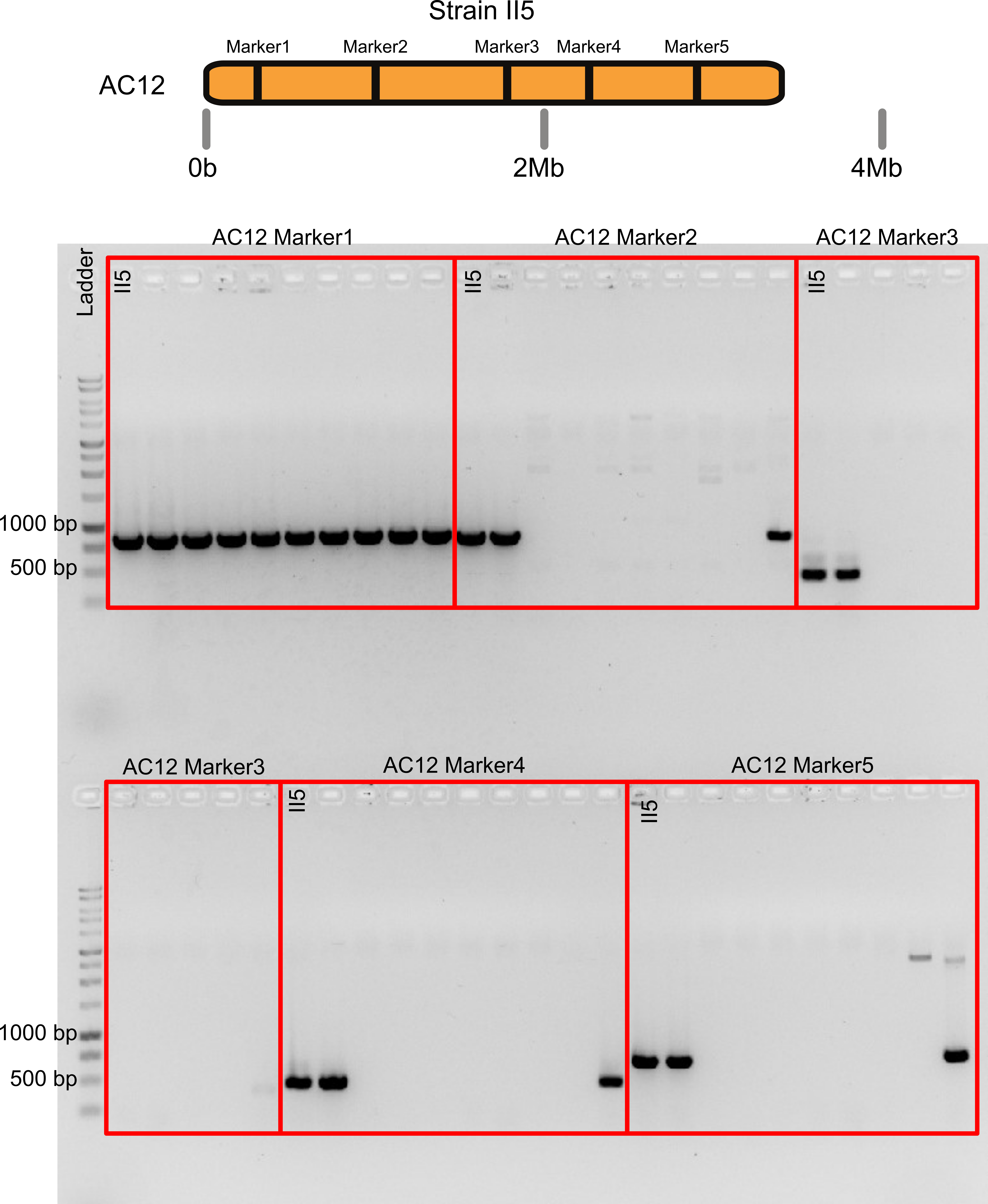


**Figure S1. - PCR analysis indicates loss of accessory chromosome 12 (AC12) after benomyl treatment. a)** Schematic representation of relative primer locations for five different AC12 PCR markers (black bands). Note that marker 1 also binds to the accessory region attached to chromosome 1. **b)** Gel electrophoresis of PCR products from the parental strain II5 and putative AC12 loss mutants. Seven hygromycin-sensitive colonies lack the bands for AC12-specific PCR markers 2-5, but still show the band for the non-specific marker 1.
