# Supplementary material for: Accessory Chromosome Contributes to Virulence of Banana Infecting Fusarium oxysporum Tropical Race 4
Source: Mol Plant Pathol. 2025 Sep 12;26(9):e70146. doi: 10.1111/mpp.70146 (PMC12430104; doi:10.1111/mpp.70146)
Supplement: Supplementary file 2 — Figure S2: Mapping of short reads to the II5 reference genome assembly. Mapping of Illumina reads from the parental strain II5 to the II5 reference genome assembly. Note the increased coverage at accessory chromosome 12 (AC12), indicative of intrachromosomal duplications. [file MPP-26-e70146-s006.docx]

**Supplementary Figures: S2**


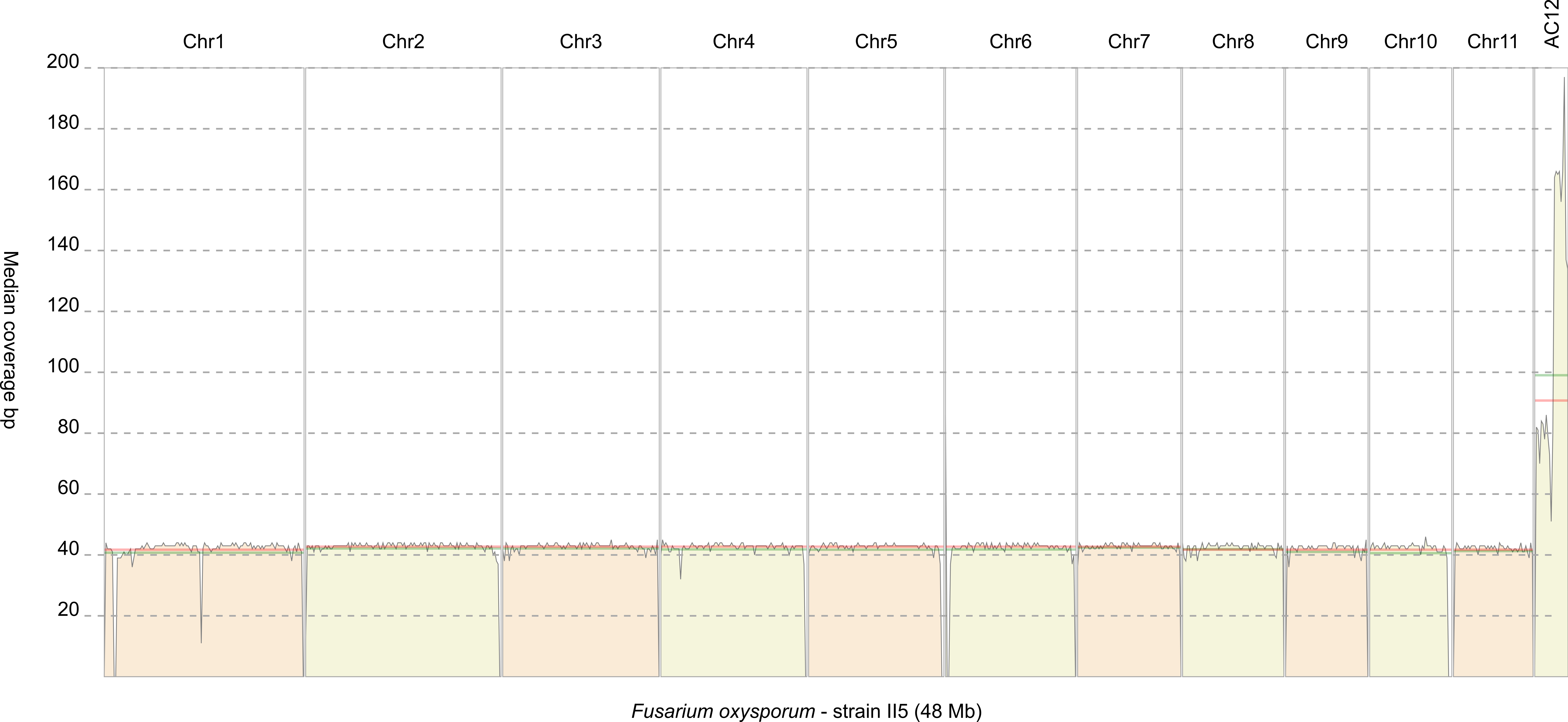


**Figure S2. - Mapping of short reads to the II5 reference genome assembly.** Mapping of Illumina reads from the parental strain II5 to the II5 reference genome assembly. Note the increased coverage at accessory chromosome 12 (AC12), indicative of intrachromosomal duplications.
