# Supplementary material for: Accessory Chromosome Contributes to Virulence of Banana Infecting Fusarium oxysporum Tropical Race 4
Source: Mol Plant Pathol. 2025 Sep 12;26(9):e70146. doi: 10.1111/mpp.70146 (PMC12430104; doi:10.1111/mpp.70146)
Supplement: Supplementary file 3 — Figure S3: AC12 mutant is vegetatively compatible with the II5 wild‐type (WT) strain. MMA plate inoculated with a nit mutant of the parental strain II5 and with a nit mutant of II5ΔAC12 7.2. Exact nitrate utilisation mutations undetermined. Annotations A and B were arbitrarily assigned to nit mutants with compatible mutations. Dense hyphal growth at the colony contact points indicates the formation of a nitrate‐utilising heterokaryon through vegetative compatibility. [file MPP-26-e70146-s007.docx]

**Supplementary Figures: S3**


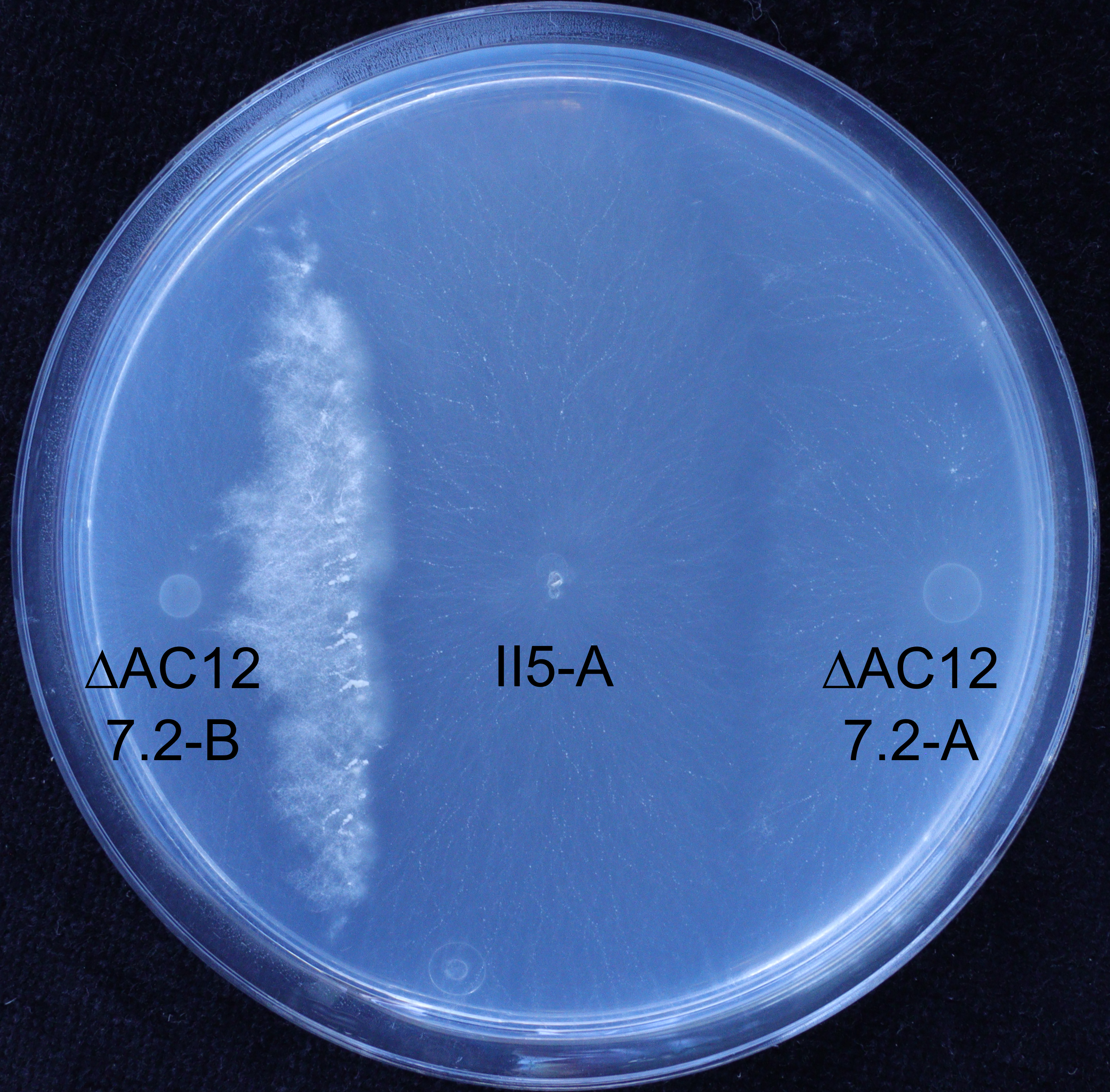


**Figure S3.** - **AC12 mutant is vegetatively compatible with the II5 WT strain.** MMA plate inoculated with a *nit* mutant of the parental strain II5 and with a *nit* mutant of II5ΔAC12 7.2. Exact nitrate utilization mutations undetermined. Annotations A and B were arbitrarily assigned to *nit* mutants with compatible mutations. Dense hyphal growth at the colony contact points indicates the formation of a nitrate-utilizing heterokaryon through vegetative compatibility.
