# Supplementary material for: Accessory Chromosome Contributes to Virulence of Banana Infecting Fusarium oxysporum Tropical Race 4
Source: Mol Plant Pathol. 2025 Sep 12;26(9):e70146. doi: 10.1111/mpp.70146 (PMC12430104; doi:10.1111/mpp.70146)
Supplement: Supplementary file 4 — Figure S4: Structural variants detected in the accessory chromosome 12 (AC12) deletion mutants. (a) The number of structural variants per variant type is shown for the seven independent AC12 deletion mutants. (b) Sizes (in bp) of the identified structural variants for the seven independent AC12 deletion mutants. Translocation sizes could not be calculated accurately due to the fragmented short read assemblies wherein translocations can be located at contig breakpoints. [file MPP-26-e70146-s008.docx]

**Supplementary Figures: S4**


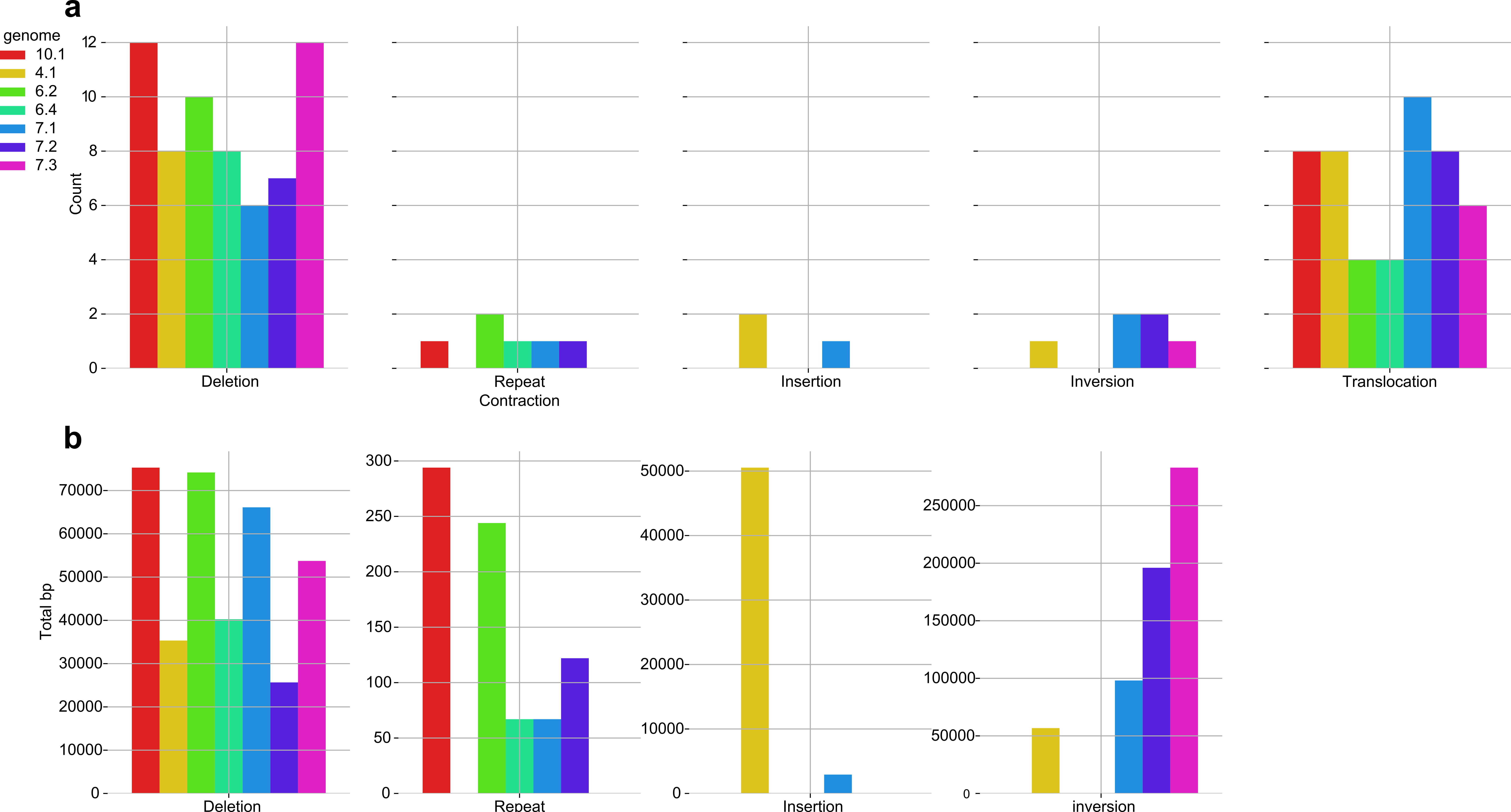


**Figure S4. - Structural variants detected in the accessory chromosome 12 (AC12) deletion mutants. a**) The number of structural variants per variant type is shown for the seven independent AC12 deletion mutants. **b**) Sizes (in bp) of the identified structural variants for the seven independent AC12 deletion mutants. Translocation sizes could not be calculated accurately due to the fragmented short read assemblies wherein translocations can be located at contig breakpoints.
