# Supplementary material for: Accessory Chromosome Contributes to Virulence of Banana Infecting Fusarium oxysporum Tropical Race 4
Source: Mol Plant Pathol. 2025 Sep 12;26(9):e70146. doi: 10.1111/mpp.70146 (PMC12430104; doi:10.1111/mpp.70146)
Supplement: Supplementary file 5 — Figure S5: Confirmation of core chromosome rearrangements in accessory chromosome 12 (AC12) loss mutant II5ΔAC12 by PCR. (a) Schematic representation of the primer locations on core chromosomes 5 and 6 in the II5 ancestor strain and the II5ΔAC12–7.2 mutant. (b) Gel electrophoresis shows PCR products from II5 and from three independent ΔAC12 mutants using different combinations of primers specific for chromosome 5 (F/R) and 6 (F/R) (see a). [file MPP-26-e70146-s001.docx]

**Supplementary Figures: S5**


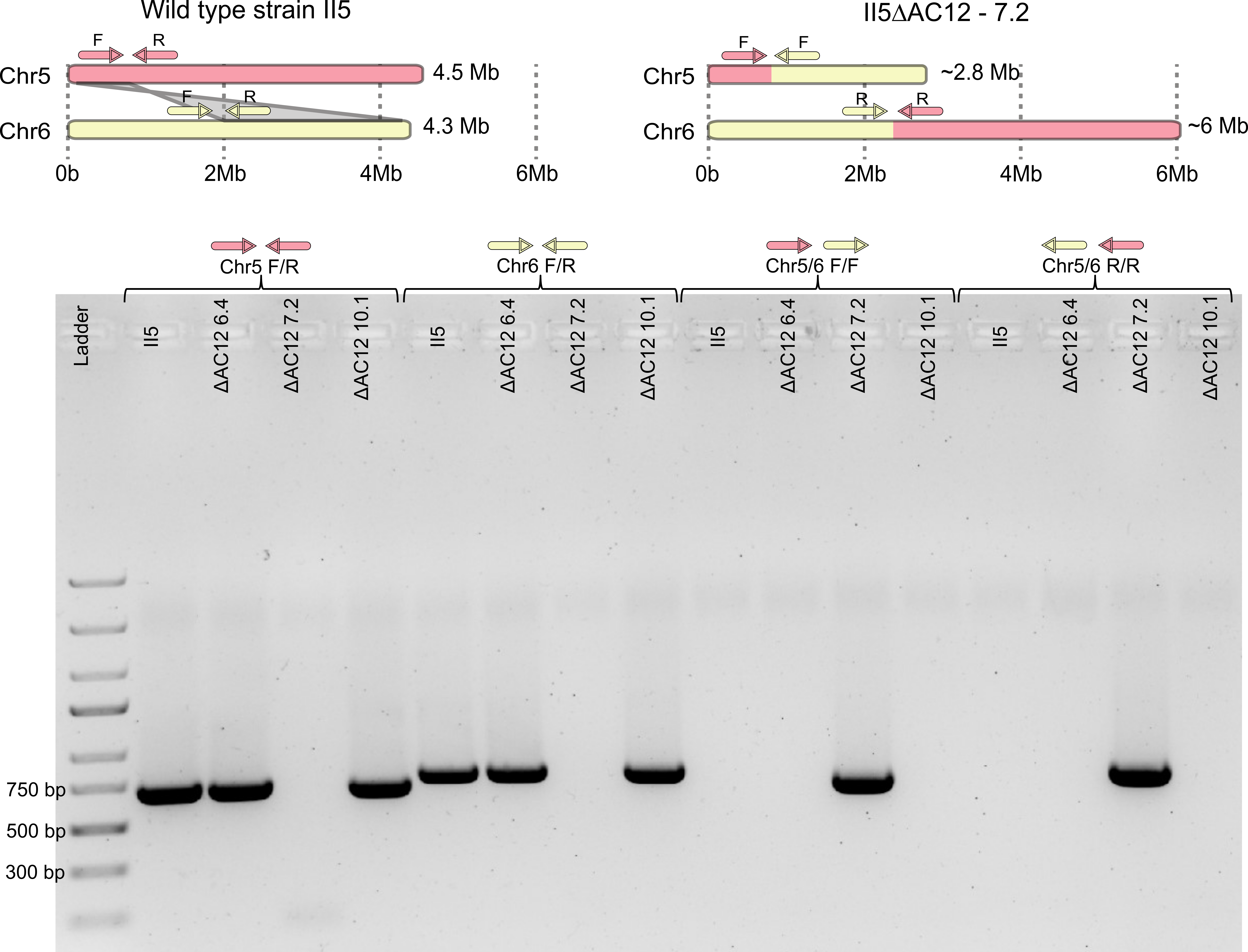


**Figure S5. Confirmation of core chromosome rearrangements in AC12 loss mutant II5ΔAC12 by PCR. a)** Schematic representation of the primer locations on core chromosomes 5 and 6 in the II5 ancestor strain and the II5ΔAC12 - 7.2 mutant. **b)** Gel electrophoresis shows PCR products from II5 and from three independent ΔAC12 mutants using different combinations of primers specific for chromosome 5 (F/R) and 6 (F/R) (see **a**).
