# Supplementary material for: Accessory Chromosome Contributes to Virulence of Banana Infecting Fusarium oxysporum Tropical Race 4
Source: Mol Plant Pathol. 2025 Sep 12;26(9):e70146. doi: 10.1111/mpp.70146 (PMC12430104; doi:10.1111/mpp.70146)
Supplement: Supplementary file 6 — Figure S6: Benomyl treatment does not affect virulence of TR4 strain II5. Percentage of corm necrosis of Cavendish ‘Grand Naine’ plants inoculated with the parental strain II5, benomyl treated control strain 3.4 and the chromosome loss strain II5ΔAC12 6.4. Corm necrosis was quantified using ImageJ (n = 10). Letters indicate significant differences between treatments (Tukey–Kramer test; p < 0.05). [file MPP-26-e70146-s005.docx]

**Supplementary Figures: S6**


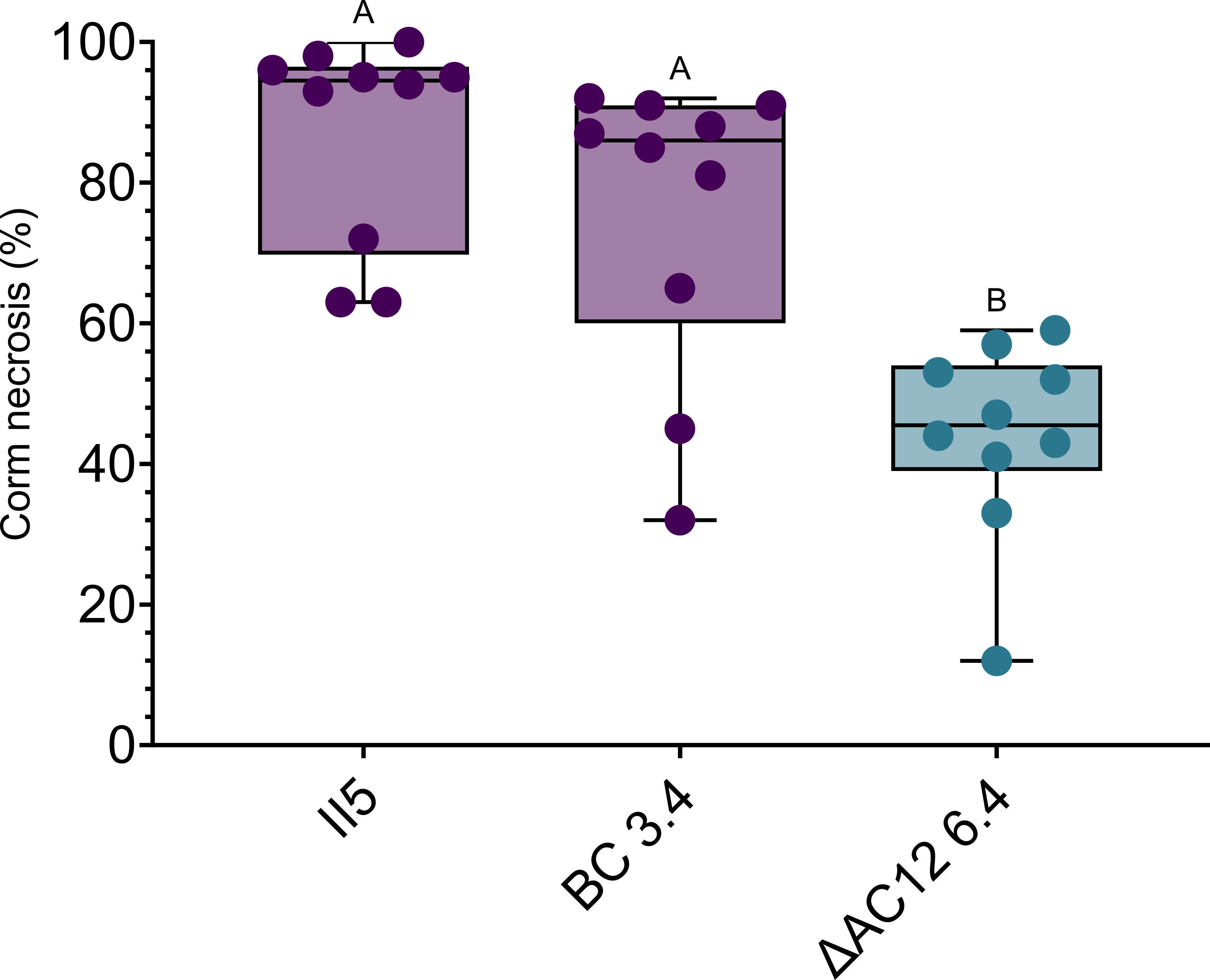


**Figure S6. -** **Benomyl treatment does not affect virulence of TR4 strain II5.** Percentage of corm necrosis of Cavendish 'Grand Naine' plants inoculated with the parental strain II5, benomyl treated control strain 3.4 and the chromosome loss strain II5ΔAC12 6.4. Corm necrosis was quantified using ImageJ (n=10). Letters indicate significant differences between treatments (Tukey-Kramer test; P<0.05).
