# Supplementary material for: Accessory Chromosome Contributes to Virulence of Banana Infecting Fusarium oxysporum Tropical Race 4
Source: Mol Plant Pathol. 2025 Sep 12;26(9):e70146. doi: 10.1111/mpp.70146 (PMC12430104; doi:10.1111/mpp.70146)
Supplement: Supplementary file 7 — Figure S7: Full uncropped CHEF gel and Southern blot used in Figure 1. [file MPP-26-e70146-s004.docx]

**Supplementary Figures: S7**





**Figure S7. -** Full uncropped CHEF gel and Southern blot used in Figure 1.
